# Two distinct Notch signals, Delta-like 4/Notch1 and Jagged-1/Notch2, antagonistically regulate chemical hepatocarcinogenesis in mice

**DOI:** 10.1038/s42003-022-03013-8

**Published:** 2022-01-21

**Authors:** Yasuhiro Nakano, Sachie Nakao, Minako Sueoka, Daigo Kasahara, Yuri Tanno, Hideaki Sumiyoshi, Tohru Itoh, Atsushi Miyajima, Katsuto Hozumi, Yutaka Inagaki

**Affiliations:** 1 Center for Matrix Biology and Medicine, Graduate School of Medicine, Tokai University, Isehara, Japan; 2 Department of Innovative Medical Science, Tokai University School of Medicine, Isehara, Japan; 3 Laboratory of Cell Growth and Differentiation, Institute for Quantitative Biosciences, The University of Tokyo, Tokyo, Japan; 4 Japan Society for the Promotion of Science, Tokyo, Japan; 5 Department of Immunology, Tokai University School of Medicine, Isehara, Japan; 6 Institute of Medical Sciences, Tokai University, Isehara, Japan

**Keywords:** Cell signalling, Cancer

## Abstract

Notch signaling is one of the most common drivers of carcinogenesis in many types of cancers, including hepatocellular carcinoma (HCC); however, it occasionally suppresses tumor progression. Moreover, it is virtually unknown how different sets of Notch ligands and receptors regulate the HCC development. In this study, we demonstrate that the expression of the Notch ligands, Delta-like 4 (Dll4) and Jagged-1 (Jag1), is upregulated during diethylnitrosamine-induced hepatocarcinogenesis. Dll4 is detected in the preneoplastic hepatocytes and HCC cells, but not in the normal hepatocytes, while Jag1 is expressed in the desmin-positive mesenchymal cells. Hepatocyte-specific *Dll4* knockout abolishes the Notch1 signaling and suppresses the tumor progression. In contrast, *Jag1* deletion induces the ectopic expression of Dll4 in hepatocytes along with the loss of Notch2 signaling, leading to the tumor progression. These results indicate that the two distinct Notch signals, Dll4/Notch1 and Jag1/Notch2, are antagonistic to each other, exerting opposite effects on HCC progression.

## Abstract (web summary)

Dll4/Notch1 signal promotes the progression of HCC, while Jag1/Notch2 signal antagonistically suppresses it in murine chemical hepatocarcinogenesis. Nakano et al. report that two distinct Notch signals regulate the progression of hepatocellular carcinoma (HCC) using tissue specific loss of function mouse mutants. They find Dll4/Notch1 signal promotes HCC progression, while the Jag1/Notch2 signal antagonistically suppresses it.

## Introduction

Hepatocellular carcinoma (HCC) is the third most common cancer worldwide^1^. In most cases, HCC develops from a pathological background of liver cirrhosis, which is a consequence of liver fibrosis caused by chronic infections with hepatitis viruses (B and C), excessive alcohol intake, and nonalcoholic steatohepatitis. The risk of developing HCC remains even after successful treatment with direct antiviral agents against the hepatitis C virus^2^. In addition, the number of patients with nonalcoholic steatohepatitis, which also leads to the development of liver cirrhosis and HCC, has been increasing in recent years^3^. Therefore, it is an urgent and strong desire to develop effective strategies to suppress the development/progression of HCC in the fibrotic liver. However, the precise molecular mechanisms underlying the pathological correlation between liver fibrosis/cirrhosis and HCC have not yet been elucidated.

Notch signaling is an evolutionarily conserved mechanism for cell-cell communication between adjacent cells^4^. Four Notch receptors (Notch 1, 2, 3, and 4) have been identified in mammalian cells. Notch ligands, including Delta-like 1 (Dll1), Delta-like 4 (Dll4), Jagged-1 (Jag1), and Jagged-2 (Jag2), bind to one or more of the Notch receptors expressed on the cell surface, resulting in proteolysis of the Notch receptor and translocation of the Notch intracellular domain (NICD) into the nucleus. The nuclear NICD later couples with the recombination signal-binding protein for immunoglobulin kappa J and mastermind-like transcriptional co-activator to control the expression of cell differentiation- and proliferation-related genes. Histological analyses of human HCC tissues suggest the potential involvement of Notch signaling in the progression of HCC^5,6^. Although many studies using murine HCC models have reported pathological correlations between HCC and Notch signaling, the specific role of Notch signaling in HCC remains controversial. For instance, overexpression of the Notch1 intracellular domain (NICD1) in hepatocytes promotes the development and progression of HCC^5^. In contrast, it has been reported that the conditional knockout of Notch1 in hepatocytes accelerates the progression of HCC via inactivation of retinoblastoma tumor suppressor pathway^7^. Furthermore, it is virtually unknown which different sets of Notch ligands and receptors promote or suppress the development and/or progression of HCC.

We have previously reported that the expression of Jag1 is upregulated in the activated hepatic stellate cells (HSCs), the major cellular source of collagen and other components of the extracellular matrix in the fibrotic liver. This ligand transduces Notch2 signaling in adjacent hepatocytes, which promotes the regeneration of fibrotic liver by accelerating the dedifferentiation and proliferation of hepatocytes^8^. Other groups have also reported that Jag1-induced Notch signaling contributes to the regeneration of the injured liver^9^. Considering that most HCCs develop from a pathological background of liver cirrhosis, it is important to examine how Jag1/Notch2 and other Notch signals regulate the progression of HCC.

In the present study, we demonstrated that the two Notch ligands, Dll4 and Jag1, exhibit antagonistic functions in the regulation of the progression of HCC. Experiments using a diethylnitrosamine (DEN)-induced hepatocarcinogenesis model^10,11^ showed that the deficiency of Dll4 inactivated Notch1 signaling and suppressed the progression of HCC. In contrast, the deletion of *Jag1* led to the ectopic expression of Dll4 in otherwise non-expressing hepatocytes with a loss of Notch2 signaling, promoting the progression of HCC. These results indicate that the two distinct sets of Notch ligands and receptors exert opposite effects on the progression of HCC. Therefore, the Jag1/Notch2 signal, which suppresses the progression of HCC while promoting the regeneration of the fibrotic liver, could be a potential therapeutic target for chronic liver disease.

## Results

### Notch signaling was activated in a murine experimental hepatocellular carcinoma model

**Fig. 1:**
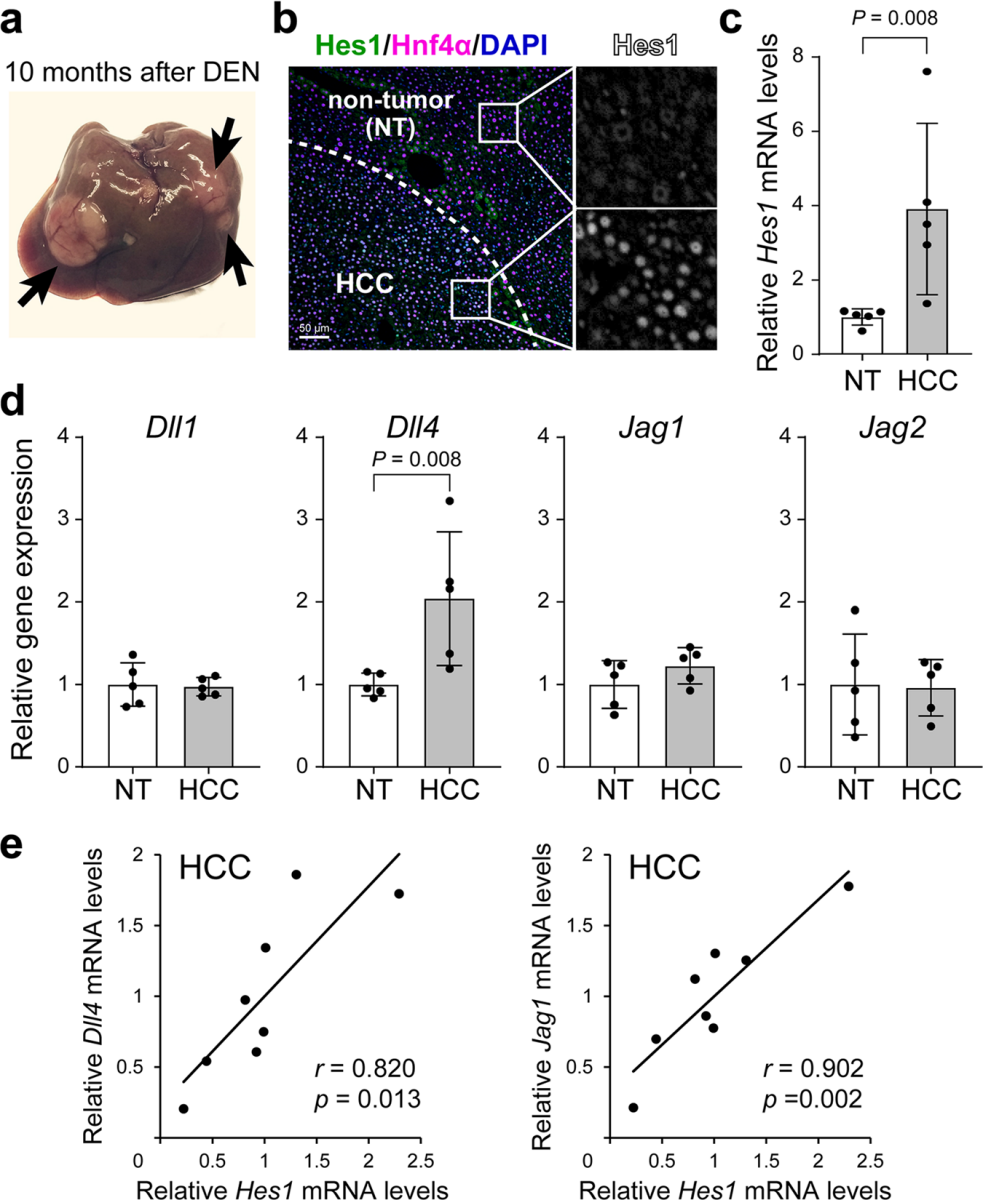
Expression levels of the Notch signal-related genes in diethylnitrosamine (DEN)-induced murine hepatocellular carcinoma (HCC). **a** A macroscopic picture of liver tumors developed 10 months after the administration of DEN. Arrows indicate the tumors observed on the liver surface. **b** DEN-induced HCC tissues were analyzed by immunofluorescent double-staining with anti-Hes1 (*green*) and anti-Hnf4α (*magenta*) antibodies together with 4′,6-diamidino-2-phenylindole (DAPI) nuclear staining (*blue*). Scale bar, 50 μm. **c**, **d** Total RNA isolated from the non-tumorous region (*NT*) and HCC was subjected to quantitative reverse-transcription (RT)-PCR analysis to detect the expression levels of *Hes1* (**c**), *Delta-like 1* (*Dll1*), *Delta-like 4 (Dll4)*, *Jagged-1* (*Jag1*), and *Jagged-2* (*Jag2*) (**d**). The values represent the mean ± standard deviation (SD) from five samples and are expressed relative to the mean value in NT (set as 1.0). **e** Correlations between the amounts of *Hes1* mRNAs and *Dll4* or *Jag1* mRNAs were analyzed using DEN-induced HCC samples obtained from eight male mice.

We generated chemically induced HCC by injecting DEN into 3-week-old male mice, as previously reported^10^. Ten months later, tumors of different sizes were observed on the surface of the liver (Fig. 1a). The tumor tissues were composed of hepatocyte nuclear factor 4 alpha (Hnf4α)-positive hepatic lineage cells, which was compatible with histopathological diagnosis of HCC. Hes1, a representative downstream target molecule of Notch signaling, was expressed in Hnf4α-positive HCC cells, but not in the Hnf4α-expressing hepatocytes present in the non-tumorous region (Fig. 1b). Consistent with this immunohistological finding, *Hes1* gene expression levels were significantly higher in the HCC tissues than the non-tumorous regions (Fig. 1c). We then identified the ligand(s) involved in the activation of Notch signaling in HCC. Among the four Notch ligands examined, only Dll4 exhibited significantly higher gene expression levels in HCC tissues than those in the non-tumorous regions (Fig. 1d). In addition, gene expression levels of *Dll4* and *Jag1* (Fig. 1e), but not those of *Dll1* and *Jag2* (Supplementary Fig. 1), were significantly correlated with those of *Hes1* in the tumors. These findings were consistent with the data available in the Gene Expression Profiling Interactive Analysis (GEPIA) database (http://gepia.cancer-pku.cn/) showing the gene expression levels of Notch ligands and their correlation with *Hes1* expression in a large number of human HCC samples (Supplementary Fig. 2). These results suggested that Dll4 and Jag1 may exert common functions in the development and progression of HCC in humans and rodents.

### Dll4 promoted the proliferation of HCC cells via Notch1 signaling

**Fig. 2:**
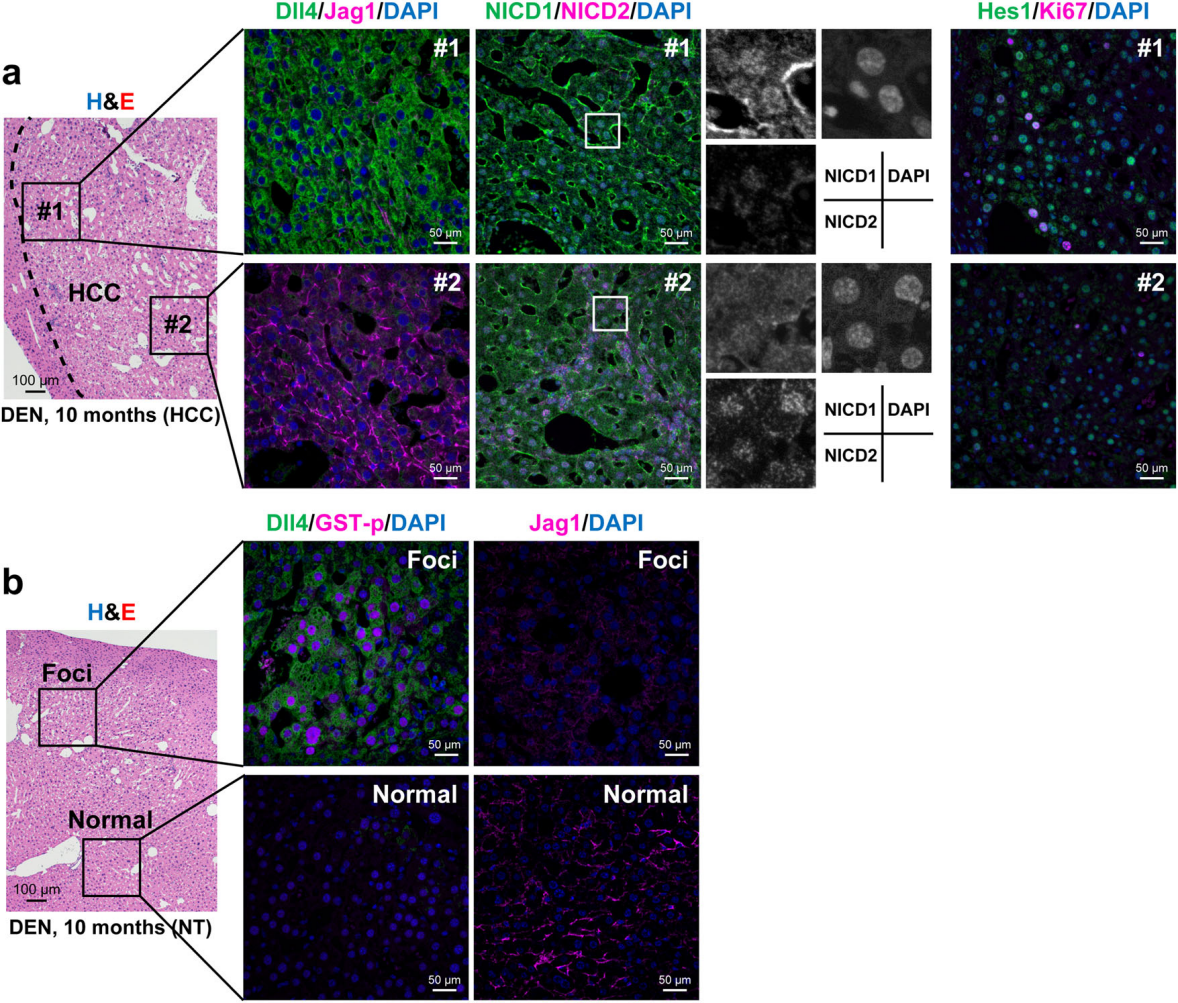
Expression levels of the Notch ligand and receptor proteins in DEN-induced HCC tissues and glutathione S-transferase placental type (GST-p)-positive preneoplastic foci. Sections prepared from HCC tissues (**a**) and preneoplastic foci (**b**) were subjected to hematoxylin and eosin (H&E) staining and immunofluorescent co-staining with antibodies against Dll4, Jag1, Notch1 intracellular domain (NICD1), Notch2 intracellular domain (NICD2), Hes1, Ki67 (a cell proliferation marker molecule), or GST-p together with DAPI nuclear staining. Two representative areas are shown that represent the peripheral (*#1*) and intrinsic (*#2*) regions within the HCC tissues. Normal liver tissues (NT) were also analyzed as controls. Scale bars, 100 μm in H&E staining and 50 μm in immunofluorescent staining.

We then examined the localization of Dll4 and Jag1 proteins in HCC tissues using immunofluorescent staining. According to the expression patterns of Dll4 and Jag1, the HCC tissues were divided into two areas (#1 and #2), which represented the peripheral and intrinsic regions of the tumor, respectively. Dll4 was strongly expressed in HCC cells present in area #1, but was hardily detected in area #2 (Fig. 2a and Supplementary Fig. 3). In contrast, Jag1 was detected in desmin-positive HSCs present in area #2, but not in cancer cells in both areas (Fig. 2a and Supplementary Fig. 3). Regarding Notch receptors, NICD1 was strongly expressed in the nuclei of cancer cells present in area #1, while the expression of Notch2 intracellular domain (NICD2) was rather weak (Fig. 2a). In contrast, NICD2, but not NICD1, was detected in the nuclei of cancer cells in area #2 (Fig. 2a). Notably, HCC cells with strong expression levels of Hes1 were predominantly observed in area #1, and some of these Hes1-expressing cancer cells were stained positive for the cell proliferation marker Ki67 (Fig. 2a). These results suggested that Dll4 promotes the proliferation of cancer cells in the peripheral region via activation of Notch1 signaling.

### Dll4 was expressed within preneoplastic foci of hepatocytes

We also determined the specific point, when the expression of Dll4 was initiated in the process of hepatocarcinogenesis. In the early phase of chemically induced hepatocarcinogenesis, preneoplastic foci are observed within the liver, which are characterized by the presence of glutathione *S*-transferase placental type (GST-p)-positive hepatocytes^12–15^. Immunofluorescent staining revealed that the hepatocytes co-expressing Dll4 and GST-p were observed in the preneoplastic foci, but not in the surrounding non-cancerous liver tissue (Fig. 2b). In contrast, Jag1 was expressed in the mesenchymal cells present in the non-cancerous region, but was hardly detected in the preneoplastic foci (Fig. 2b). These staining patterns were similar to those observed in the peripheral regions of HCC, indicating that Dll4 is a key molecule in the early phase of carcinogenesis in the liver.

### Dll4-induced Notch1 signaling promoted the progression of HCC

**Fig. 3:**
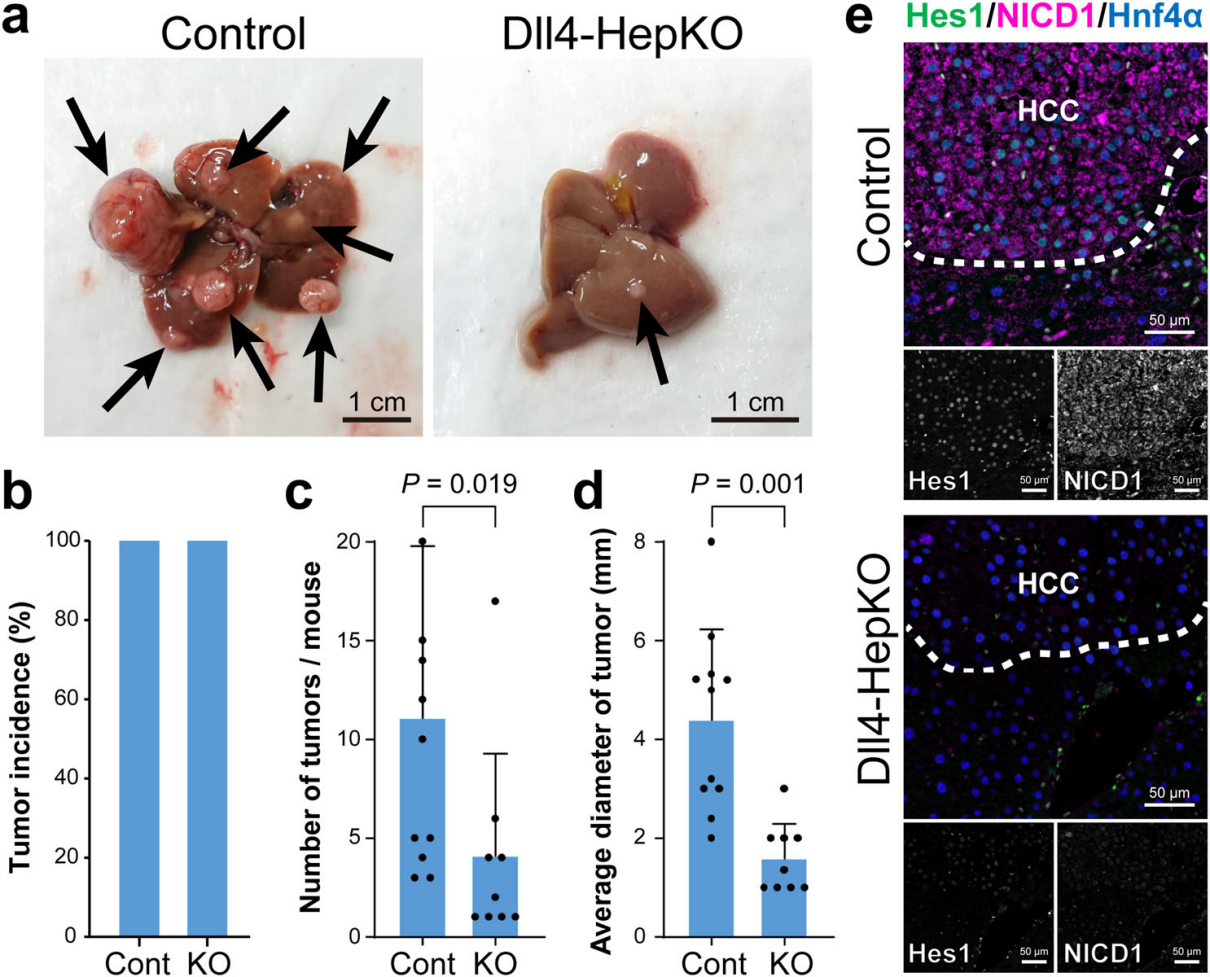
Development of HCC suppressed by the hepatocyte-specific deletion of *Dll4*. **a** Representative macroscopic pictures of liver tumors observed in DEN-treated male control or hepatocyte-specific Dll4 knockout mice (Dll4-HepKO). Arrows indicate the tumors observed on the liver surface. The histograms show the incidence of development of tumors (**b**), the mean number of tumors (**c**), and the average size of tumors (**d**) observed in the control (Cont) and Dll4-HepKO (KO) mice. The values represent the mean ± SD in 11 samples from the control mice and nine samples from the Dll4-HepKO mice. **e** Excised HCC specimens were subjected to immunofluorescent co-staining using antibodies against Hes1 (*green*), NICD1 (*magenta*), and Hnf4α (*blue*). The border between HCC and non-tumorous tissue is indicated by a hatched line. Scale bar, 50 μm.

To further understand the role of Dll4 in the progression of HCC, we conducted hepatocyte lineage-specific *Dll4* deletion (Dll4-HepKO) including HCC cells. For this purpose, DEN-treated *Dll4*^*loxP*/*loxP*^ male mice, at 10 weeks of age, were injected with adeno-associated virus capsid 8 (AAV8) that expressed the *iCre* gene driven by a hepatocyte-specific promoter (AAV8-LSP-iCre)^16^. Ten months after the administration of DEN, HCC was found to be developed in both Dll4-HepKO mice and control animals (Fig. 3a, b and Supplementary Fig. 4a). However, Dll4-HepKO mice had significantly fewer tumors on the surface of their livers compared to the control mice (Fig. 3c). Furthermore, the mean size of tumors was significantly smaller in Dll4-HepKO mice than in the control animals (Fig. 3d). In Dll4-deficient HCC tissues, the expression levels of NICD1 and Hes1 were remarkably diminished (Fig. 3e). In contrast, *Dll4* deletion did not affect the expression of Jag1 or the activation of Notch2 signaling (Supplementary Fig. 5). Collectively, these results indicated that the expression of Dll4 in hepatocyte-lineage cells activates the Notch1 signaling and promotes the progression of DEN-induced HCC.

### Jag1 suppressed the progression of chemically induced HCC

**Fig. 4:**
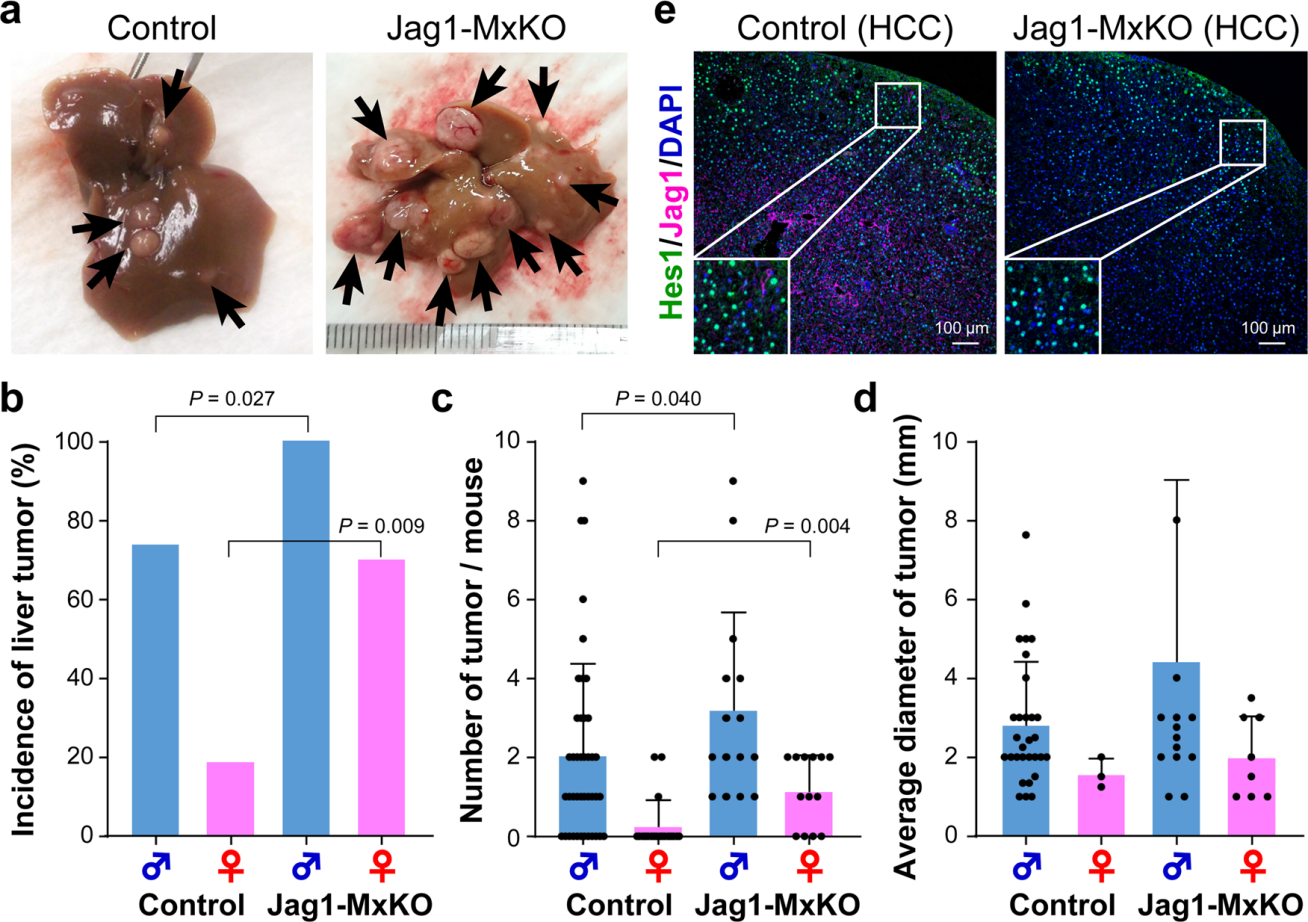
Development of HCC induced by the deletion of *Jag1*. **a** Representative macroscopic pictures of liver tumors observed in DEN-treated male control or Jag1 conditional knockout mice (Jag1-MxKO). Arrows indicate the tumors observed on the liver surface. The histograms show the incidence of development of tumors (**b**), the mean number of tumors (**c**), and the average size of tumors (**d**) observed in the control and Jag1-MxKO male and female mice. The values represent the mean ± SD from the control (42 males and 19 females) and Jag1-MxKO (15 males and 11 females) mice. **e** Excised HCC specimens were subjected to immunofluorescent co-staining using anti-Hes1 (*green*) and anti-Jag1 antibodies (*magenta*) together with DAPI nuclear staining (*blue*). Scale bar, 100 μm.

We also investigated the role of another Notch ligand, Jag1, in the progression of HCC. DEN-treated *Mx-Cre*/*Jag1*^*loxP*/*loxP*^ male and female mice were administered polyinosinic-polycytidylic acid [poly(I:C)]^17^ four times at 8–10 weeks of age (Jag1-MxKO). It is known that the development of DEN-induced HCC is inhibited by poly(I:C)-induced type I interferon^18^. Accordingly, the incidence of HCC in the control male mice (74%) was lower than that in the control male mice (100%) shown in the above Dll4-knockout experiment (Fig. 4a, b and Supplementary Fig. 4b). In addition, consistent with the finding that estrogen-mediated interleukin 6 suppresses the development of HCC^19^, the incidence of HCC in the control female mice was found to be only 16% in the present study (Fig. 4a, b). Moreover, *Jag1* deletion caused a significant increase in the incidence of HCC both in the male (100%) and female (69%) mice compared with that of the controls (Fig. 4b). The number of tumors was also increased significantly in both male and female Jag1-MxKO mice compared with the control animals (Fig. 4c), but there was no significant difference in the mean tumor size between the two groups (Fig. 4d). Immunostaining of HCC tissues from Jag1-MxKO mice confirmed a complete loss of Jag1 expression, but Hes1 expression remained unchanged, especially in the peripheral regions of the tumor (Fig. 4e). Deletion of *Jag1* did not affect the expression of Dll4 or the activation status of Notch1, but suppressed the nuclear translocation of NICD2 (Supplementary Fig. 6). These results indicated that Jag1/Notch2 signaling suppresses the development of chemically induced HCC.

### *Jag1* deletion led to a loss of Notch2 signaling and induced the ectopic expression of Dll4 in hepatocytes

**Fig. 5:**
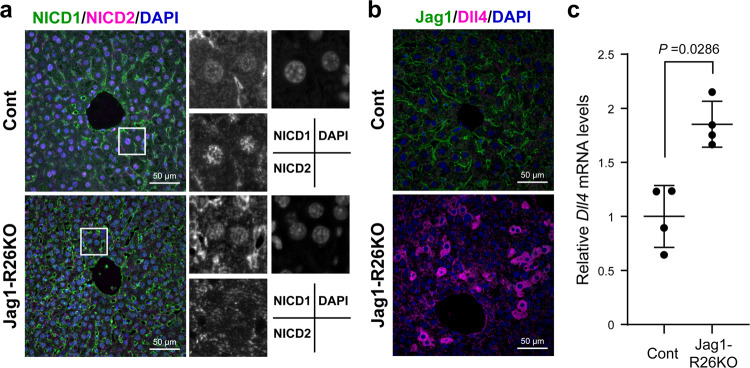
Ectopic expression of Dll4 in hepatocytes induced by the loss of Jag1/Notch2 signaling. (**a**, **b**) Immunofluorescent co-staining of NICD1 (*green*) and NICD2 (*magenta*) (**a**) or Jag1 (*green*) and Dll4 (*magenta*) (**b**) was performed using the liver tissues from the DEN-treated male control (Cont) and *Rosa26*^*CreER*/+^/ *Jag1*^*loxP*/*loxP*^ (Jag1-R26KO) mice that were administered tamoxifen at 8 weeks of age. Nuclei were stained with DAPI (*blue*). Scale bar, 50 μm. **c** Quantitative RT-PCR analysis of *Dll4* gene was performed using liver tissue from male control and Jag1-R26KO mice. The values represent the mean ± SD from four samples in each group and are expressed relative to the mean value in control (set as 1.0).

To avoid any immunological influence of the poly(I:C) injections on the development of HCC, we also evaluated the effect of *Jag1* deletion using another mouse strain, *CreER* knock-in mice, at the ubiquitous promoter *Rosa26* locus (*Rosa26*^*CreER*/+^)^20^. Jag1 deficiency was induced in DEN-injected *Rosa26*^*CreER*/+^/*Jag1*^*loxP*/*loxP*^ mice (Jag1-R26KO) by intraperitoneal injection of tamoxifen four times every other day. In the control mouse liver, both NICD1 and NICD2 were observed in hepatocyte nuclei (Fig. 5a). However, the expression levels of NICD2 were remarkably diminished in the Jag1-R26KO hepatocytes (Fig. 5a), indicating that Notch2 signaling depends on Jag1. More importantly, *Jag1* deletion induced the ectopic expression of Dll4 in hepatocytes (Fig. 5b, c). Experiments using primary cultures of hepatocytes indicated that *Dll4* gene expression was suppressed by coating the culture dishes with Jag1-Fc chimera, which was restored by adding a Notch signaling inhibitor, gamma secretase inhibitor-IX (Supplementary Fig. 7a). Collectively, these results indicated that the Jag1/Notch2 signaling prevents the progression of HCC by suppressing the expression of Dll4 and Dll4-mediated Notch1 signaling.

### Notch1 and Notch2 signals exerted opposite effects on the proliferation and apoptosis of hepatocytes

**Fig. 6:**
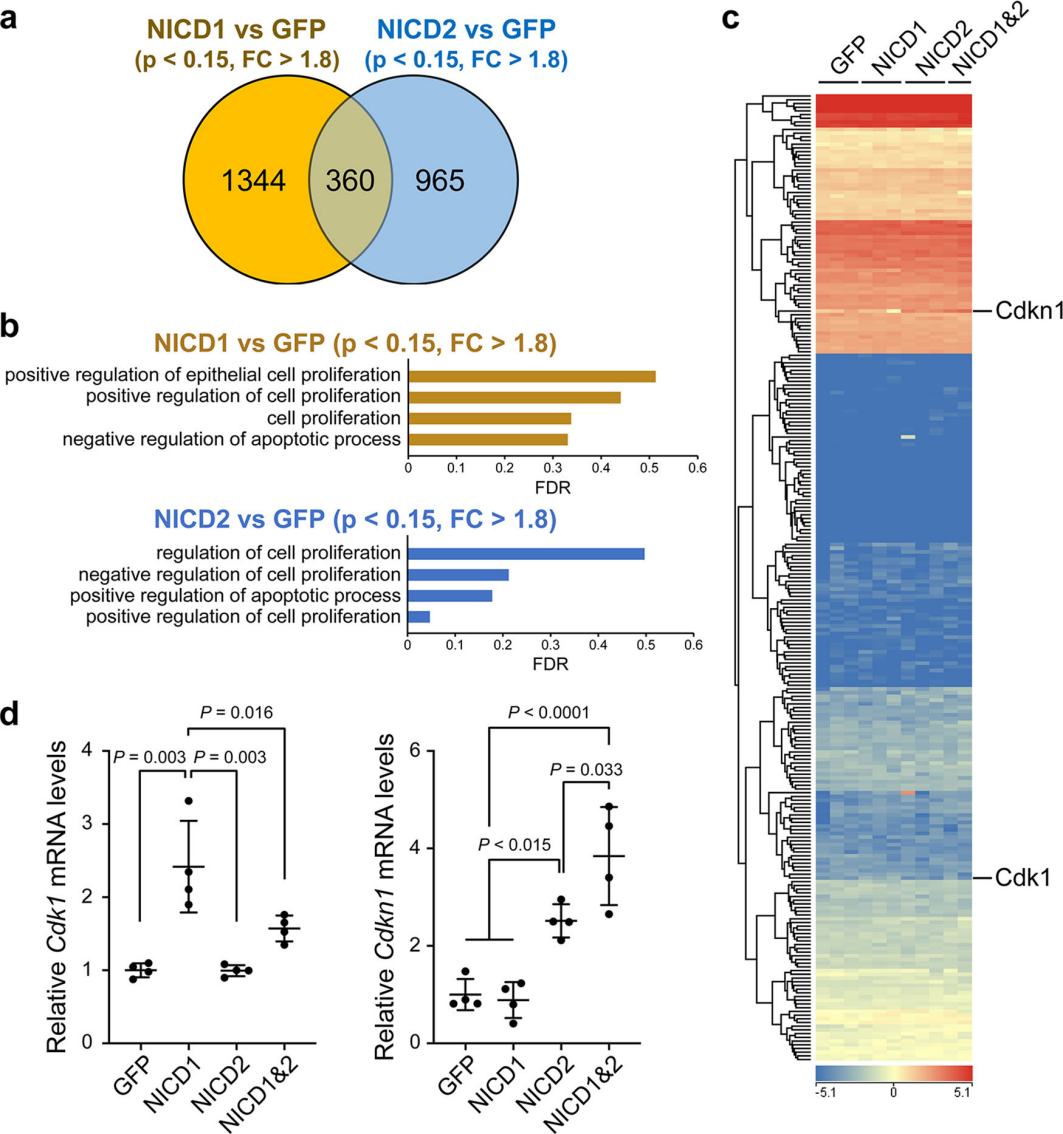
Comprehensive gene expression analysis in liver tissues following forced activation of the Notch1 and Notch2 signaling. C57Bl6/J male mice were intraperitoneally injected with adeno-associated virus 8 (AAV8) expressing the control green fluorescent protein (GFP) (6 × 10^11^ viral genomes (vg) per mouse), NICD1 and GFP (3 × 10^11^ vg each per mouse), NICD2 and GFP (3 × 10^11^ vg each per mouse), or NICD1 and NICD2 (3 × 10^11^ vg each per mouse). Five days later, total RNA was isolated from the excised liver tissues and subjected to expression microarray analysis (**a**–**c**) and quantitative RT-PCR (**d**). The Venn diagram (**a**) and enriched Gene Ontology (GO) terms (**b**) indicate the changes in the gene expression profiles following the overexpression of NICD1 or NICD2 compared to the overexpression of the control GFP. The differentially expressed genes were defined by *P* <  0.15 and a fold change (FC) >1.8 (*n* = 3 in each group). **c** Heatmap illustrating the expression levels of the cell proliferation-related genes annotated in GO analysis, ‘G2/M transition of mitotic cell cycle’ (GO:0000086) in the liver (*n* = 3 in GFP, NICD1, and NICD2 groups; *n* = 2 in NICD1&2 group). **d** Quantitative RT-PCR analysis of the selected candidate genes. The values represent the mean ± SD from four mice in each group and are expressed relative to those in the control GFP group (set as 1.0).

In the last set of experiments, we compared the effects of Notch1 and Notch2 signaling induced by AAV8-mediated forced expression of NICD1 and NICD2, respectively, in the liver. Eight-week-old wild-type male mice were intraperitoneally injected with an NICD1- and/or NICD2-overexpressing AAV8 vector. Efficient AAV8-mediated gene transfer was confirmed by the fluorescence of the co-expressed green fluorescent protein (GFP) in hepatocytes (Supplementary Fig. 8). A comprehensive gene expression analysis using DNA microarray indicated that the expression levels of 1704 and 1325 genes were altered by overexpressing NICD1 and NICD2, respectively, compared with the control group that only expressed GFP (Fig. 6a). Gene ontology analysis revealed the enrichment of cell proliferation accelerators and anti-cell apoptotic pathways in the NICD1 group, while the NICD2 group was enriched with genes that suppress the cell proliferation and promote apoptosis (Fig. 6b). Heat map analysis indicated that the cell proliferation gene, cyclin-dependent kinase 1 (*Cdk1*), and its inhibitory factor gene, *Cdkn1* (p21), were reciprocally increased by overexpressing NICD1 and NICD2, respectively (Fig. 6c). Quantitative reverse-transcription (RT)-PCR further evaluated the changes in the expression levels of *Cdk1* and *Cdkn1* genes. Increased *Cdk1* expression by forced NICD1 expression was counter-suppressed by co-expressing NICD2, while *Cdk1* expression was significantly increased by the forced expression of NICD2 (Fig. 6d). These results clearly indicated that Notch1 and Notch2 signals are antagonistic to each other and exert opposite effects on the proliferation and apoptosis of hepatocytes.

## Discussion

**Fig. 7:**
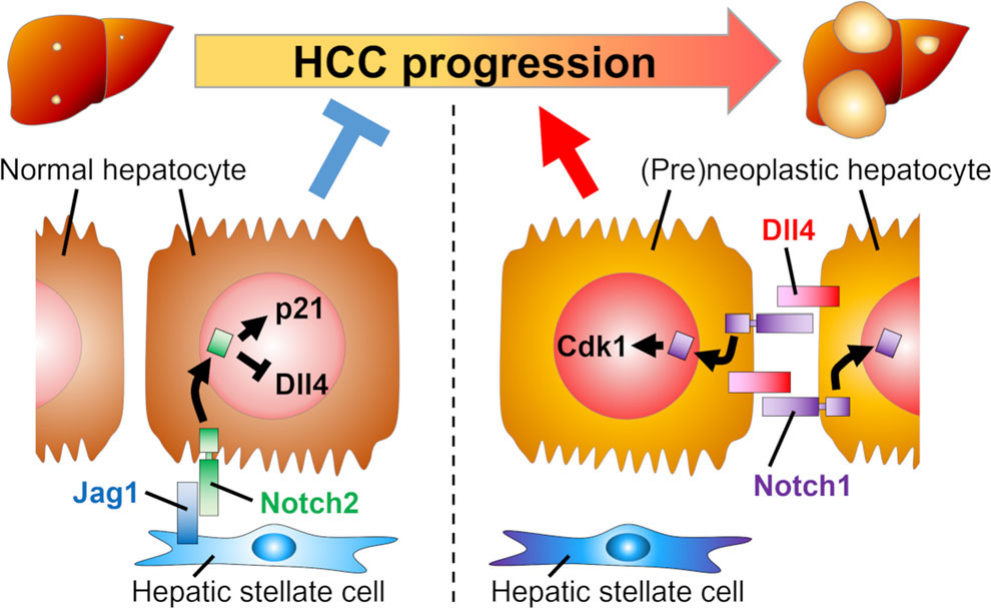
Schematic illustration of the two distinct Notch signals in HCC progression. Dll4/Notch1 signal promotes the progression of HCC, while Jag1/Notch2 signal antagonistically suppresses it in murine chemical hepatocarcinogenesis.

In the present study, we have shown that Dll4 and Jag1 exhibit antagonistic effects on the progression of HCC. Dll4 is expressed in cancer cells and activates Notch1 signaling in an autocrine manner, while Jag1 is expressed in the neighboring HSCs and activates Notch2 signaling in adjacent cancer cells. Experiments using conditional knockout mice indicated that hepatocyte lineage-specific *Dll4* deletion abolished the Notch1 signaling and suppressed the progression of HCC. On the other hand, *Jag1* deletion induced the ectopic expression of Dll4-Notch1 in the hepatocytes with a loss of Notch2 signaling, leading to the progression of HCC. Furthermore, forced NICD1 expression increased the expression of *Cdk1* and stimulated the proliferation of hepatocytes, while overexpression of NICD2 counter-suppressed this stimulatory effect by inducing the expression of *Cdkn1* coding for p21, a well-known inhibitor of the proliferation of hepatocytes^21^. These results clearly indicate that different combinations of Notch ligands and receptors exert distinct effects on the progression of HCC (Fig. 7).

The distinct functions of Notch ligands through their binding to different receptors have been well illustrated in the process of lymphocyte differentiation. Dll4, but not Jag1 or Dll1, is essential for the differentiation of T cells in the thymus via activation of Notch1 signaling in the hematopoietic progenitor cells^22–24^. On the other hand, Dll1 induces the differentiation of splenic B cells into the marginal zone B cells via the activation of Notch2 signaling^25,26^. In contrast to these findings in hematopoietic organs, little is known about the functional differences among the distinct sets of Notch ligands and receptors in the liver. A combination of Jag1 and Notch2 was originally implicated in the regulation of the differentiation of HPCs into biliary epithelial cells and the formation of the ductal structure during the development of fetal liver^27,28^. In healthy liver regeneration, the expression of Notch1 increases in hepatocytes and the activation of Notch1 signaling accelerates hepatocyte proliferation following partial hepatectomy^29–31^. In addition, we previously reported that Jag1/Notch2 signaling accelerated the regeneration of the fibrotic liver by inducing the dedifferentiation of hepatocytes into an HPC population^8^. However, it was virtually unknown before the present study whether specific combinations of Notch ligands and receptors play critical roles in hepatocarcinogenesis.

A previous study using a mouse model of HCC and cholangiocellular carcinoma (CCC) induced by the overexpression of v-Akt and N-Ras oncogenes in hepatocytes reported the different effects of the blockade of Notch1, Notch2, and Notch3 signaling on the tumor progression^32^. It was found that the administration of anti-Notch2 antibodies suppressed the development of HCC/CCC, while the administration of anti-Notch3 antibodies exhibited no such effects on tumor progression. Interestingly, blockade of Notch1 signaling altered the relative prevalence of HCC/CCC, suppressing the development of HCC and promoting the occurrence of CCC. However, these findings contradict the results of the present study regarding the effect of Notch2 signaling on the development of HCC. In addition to the different models (viral oncogenes versus chemical carcinogen) and methods (neutralizing antibodies against Notch receptors versus deletion of ligand genes and overexpression of the NICDs) employed, the discrepancy between the results of these two studies illustrates the complex interactions between different Notch ligands and receptors in the context of tumor development and progression. It would be interesting to know whether the differential roles of Notch1 and Notch2 signals are observed in viral oncogene-induced HCC by utilizing the same loss-of-function experiments as in the present study.

It should be noted that the ectopic expression of Dll4 was observed in the GST-p-positive hepatocytes within the preneoplastic foci, but not the normal hepatocytes present in the surrounding non-cancerous tissues (Fig. 2b). In contrast, Jag1 was expressed in the mesenchymal cells in the non-cancerous region, but not in cells within the preneoplastic foci (Fig. 2b). These findings suggest a signal switch from Jag1 to Dll4 in the very early phase of carcinogenesis in the liver. Jag1 expression is reportedly induced by Wnt/β-catenin signaling^33^. The results of the present study showed that the expression levels of the *Dll4* gene in the primary cultures of hepatocytes were suppressed by the activation of Wnt/β-catenin signaling (Supplementary Fig. 7b). Therefore, the Wnt/β-catenin axis might be one of the possible pathways that regulates the reciprocal expression of Jag1 and Dll4 during hepatic carcinogenesis. However, the precise mechanisms that determine the combination of Notch ligands and receptors during hepatic carcinogenesis are still unknown. The fringe molecule-induced glycosylation of Notch receptors increases their binding to Dll1 and Dll4, while decreasing their binding to Jag1 and Jag2^34^. These changes in the relative affinities of Dll4 and Jag1 to the Notch receptors determine the intensities of downstream signals. Therefore, fringe-induced glycosylation may modulate the progression of HCC. Our preliminary data indicated that the lunatic fringe was expressed in those HCC cells that exhibited a remarkable activation of Notch1 signaling. Further studies are needed to explore the correlation between the glycosylation of the Notch receptors and the progression of HCC.

HCC develops not only in the progressive phase of liver cirrhosis but also in the recovery period after sustained virologic response to interferons and direct anti-viral agent therapy against chronic hepatitis C and cirrhosis^2^. Increased Jag1 expression in activated HSCs^8^ may decrease along with the gradual improvement in the extent of fibrosis during this recovery phase. Based on these findings, the induction/retention of Jag1 expression even after a sustained virologic response might be an effective intervention strategy to prevent the occurrence of HCC. Moreover, patients with HCC exhibiting a high Wnt/β-catenin signal activity have been reported to present a better prognosis than those with low Wnt/β-catenin activity^35,36^. In addition, Notch signaling and Wnt/β-catenin signaling exert antagonistic effects on the regeneration of chronically injured liver^9^. Taken together, Wnt/β-catenin signal-elicited suppression of Dll4 in hepatocytes and induction of Jag1in HSCs may suppress the progression of HCC, while accelerating the regeneration of fibrotic liver.

## Materials and methods

### Mice

All mice used in the present study were handled with care, and the animal experimental procedures were carried out in accordance with the National Institutes of Health Guide for the Care and Use of Laboratory Animals. The experiment was approved by the Animal Experimentation Committee of Tokai University (approval Nos. 181031, 181066, 192035, and 201021). The C57BL/6 J mice were purchased from CLEA Japan Inc. (Tokyo, Japan). For the hepatocyte-specific *Dll4* knockout, *Dll4*-floxed knock-in male mice (*Dll4*^*lox*/*lox*^)^22^, at the age of 8 weeks, were injected intraperitoneally with 1 × 10^11^ viral genomes per mouse of AAV8-LSP-iCre^16^, which expresses the *iCre* gene under the control of the human apolipoprotein E enhancer and the alpha 1-antitrypsin promoter. AAV8-LSP-iCre was prepared by transfecting AAV8-LSP-iCre^16^, p5E18-VD2/8^16^, and XX680 plasmids^16^ into AAVpro293T cells (Takara Bio, Ohtsu, Japan)^37^. *Jag1*-floxed knock-in (*Jag1*^*lox*/*lox*^)^33^ mice were crossed with *Mx-Cre* transgenic mice^17^ or *Rosa26*^*CreER*/+^ mice^20^ that had been backcrossed with the C57BL/6 J mice. To achieve *Jag1* deletion, *Mx-Cre*/*Jag1*^*loxP*/*loxP*^ mice and the control *Mx-Cre*-negative *Jag1*^*lox*/*lox*^ littermates were administered four injections of 250 μg of poly(I:C) (Sigma-Aldrich, St. Louis, MO) every 3 days at the age of 8 weeks. For the same purpose, *Rosa26*^*CreER*/+^/*Jag1*^*loxP*/*loxP*^ mice were intraperitoneally injected with 100 mg per kg body weight of tamoxifen (Sigma-Aldrich) four times every other day. DEN (Sigma-Aldrich) was dissolved in saline and injected intraperitoneally (10 mg per kg body weight) on postnatal day 21.

### Histological examination

Excised liver tissues were fixed with 4% paraformaldehyde, dehydrated, and then embedded in a paraffin block. Tissue sections of 2 μm thickness were prepared using a microtome. The sections were stained with hematoxylin and eosin (H&E) using standard protocols^38^. For immunofluorescence staining, the deparaffinized sections were soaked in Target Retrieval Solution (pH 9.0) (Dako, Glostrup, Denmark) and autoclaved at 110 °C for 10 min. After inactivation of endogenous peroxidase and blocking of non-specific protein-binding, the sections were incubated at room temperature for 2 h with the specific primary antibodies listed in Supplementary Table 1. Bound primary antibodies were visualized using fluorescent secondary antibodies (Supplementary Table 2) and the sections were examined under a fluorescence microscope (BZ-9000; Keyence Corp., Osaka, Japan). Nuclei were stained with 4′,6-diamidino-2-phenylindole (Sigma-Aldrich).

### Isolation and primary cultures of hepatocytes

Hepatocytes were isolated from the murine liver using the collagenase perfusion method^8^ and subjected to primary culture on a bovine type I collagen-coated dish (AGC TECHNO GLASS, Shizuoka, Japan). In some experiments, primary hepatocytes were cultured in dishes precoated with recombinant human Jagged-1 Fc-chimera protein (R&D Systems, Minneapolis, MN). Gamma secretase inhibitor IX (DAPT; Merck, Darmstadt, Germany) was diluted in dimethyl sulfoxide (Sigma-Aldrich) before use.

### Quantitative RT-PCR

Total RNA was isolated from the liver tissues or cultured cells using the RNeasy Plus Mini Kit (Qiagen, Hilden, Germany), according to the manufacturer’s instructions. Subsequently, the RNA was reverse transcribed using the ReverTra Ace qPCR RT Master Mix with gDNA remover (TOYOBO, Osaka, Japan). Quantitative PCR was then performed using the SYBR Green PCR Master Mix (Applied Biosystems, Foster City, CA) with specific primers listed in Supplementary Table 3.

### Generation of AAV8 vectors overexpressing NICD1 and NICD2

NICD1 and NICD2 cDNA sequences were excised from pMY-NICD1-IRES-GFP and pMY-NICD2-IRES-GFP, respectively^39^, and cloned into the pAAV-CMV vector (Takara Bio) to generate pAAV-CMV-NICD1 and pAAV-CMV-NICD2, respectively. pAAV-CMV-GFP^40^ was used as a control vector. Recombinant AAV8 viruses were prepared by co-transfecting pAAV-CMV-NICD1 or pAAV-CMV-NICD2 together with p5E18-VD2/8 and pHelper (Takara Bio) into AAVpro293T cells using the AAVpro Purification Kit (Takara Bio). The viral titers were determined by quantitative PCR using an AAVpro Titration Kit Ver.2 (Takara Bio). Subsequently, the 8-week-old male C57BL/6J mice were intraperitoneally injected with 3 × 10^11^ viral genomes of AAV8 viruses.

### Microarray gene expression analysis

Total RNA was prepared from liver tissue using the RNeasy Micro Kit (Qiagen). Gene expression profiles were analyzed using the Whole Mouse Genome Microarray 8 × 44 K (Agilent Technologies, Santa Clara, CA). Hierarchical clustering of normalized signal intensities was performed using Euclidean distances and centroid linkages. Raw intensity values were normalized using the 75th percentile and transformed to the log2 scale. The original microarray data were deposited in the Gene Expression Omnibus (accession number: GSE178886).

### Statistics and reproducibility

All experiments were independently repeated using at least three mice per experimental group. Values are expressed as the mean ± standard deviation. Statistical analyses were performed using Microsoft Excel 2013 (Microsoft, Seattle, WA) and GraphPad Prism 8 (GraphPad Software Inc., San Diego, CA). Significant correlations were estimated using the Pearson product-moment correlation coefficient and log-rank test. Statistical differences between groups were evaluated using either the Chi-squared test, Mann–Whitney *U* test (two-tailed), or One-Way ANOVA (two-tailed), and *P* values <0.05 were considered statistically significant.

### Reporting summary

Further information on research design is available in the Nature Research Reporting Summary linked to this article.

## Supplementary information

Supplementary Information

Description of Additional Supplementary Files

Supplementary Data 1

Reporting Summary

## Data Availability

Source data for the graph figures are available in Supplementary Data 1. The microarray data for this study have been deposited in GEO at NCBI accession number GSE178886.
