# Supplementary material for: Two distinct Notch signals, Delta-like 4/Notch1 and Jagged-1/Notch2, antagonistically regulate chemical hepatocarcinogenesis in mice
Source: Commun Biol. 2022 Jan 21;5:85. doi: 10.1038/s42003-022-03013-8 (PMC8782997; doi:10.1038/s42003-022-03013-8)
Supplement: Supplementary file 1 — Supplementary Information [file 42003_2022_3013_MOESM1_ESM.pdf]

Supplementary Information

Two distinct Notch signals, Delta-like 4/Notch1 and Jagged-1/Notch2, antagonistically regulate chemical hepatocarcinogenesis in mice

Yasuhiro Nakano, Sachie Nakao, Minako Sueoka, Daigo Kasahara, Yuri Tanno, Hideaki Sumiyoshi, Tohru Itoh, Atsushi Miyajima, Katsuto Hozumi, and Yutaka Inagaki

Supplementary Figure 1. Correlations between the amounts of *Hes1* mRNAs and four Notch ligand mRNAs in diethylnitrosamine (DEN)-induced murine hepatocellular carcinoma (HCC) samples.

Supplementary Figure 2. Expression levels of Notch ligands in human HCC tissues.

Supplementary Figure 3. Differential expression of Dll4 and Jag1 in DEN-induced murine HCC.

Supplementary Figure 4. Histological features of HCC tissues developed in Dll4-HepKO and Jag1-MxKO mice.

Supplementary Figure 5. Expression of Jag1 and activation status of Notch2 in cancer cells induced by *Dll4* deletion.

Supplementary Figure 6. Expression of Dll4 and activation status of Notch1/Notch2 in cancer cells induced by *Jag1* deletion.

Supplementary Figure 7. Effects of Notch and Wnt signaling on the expression levels of *Dll4* in the primary cultures of hepatocytes.

Supplementary Figure 8. Efficient infection of AAV8 to hepatocytes in the liver.

Supplementary Table 1. Primary antibodies.

Supplementary Table 2. Secondary antibodies.

Supplementary Table 3. Primers used for quantitative RT-PCR.

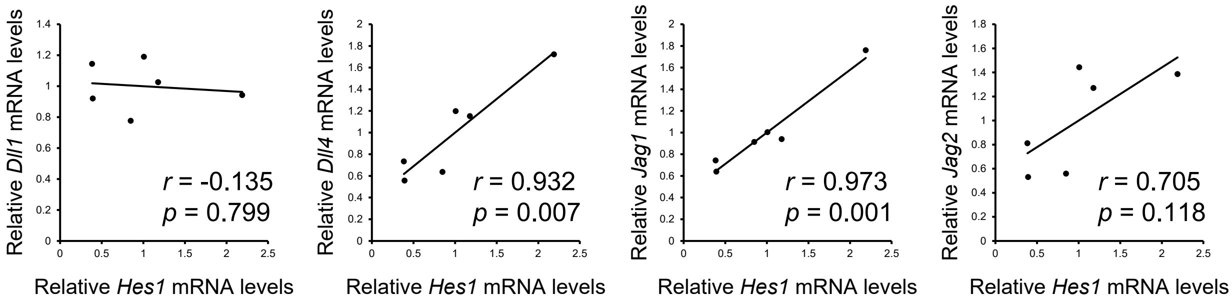

Supplementary Figure 1. Correlations between the amounts of Hes1 mRNAs and four Notch ligand mRNAs in diethylnitrosamine (DEN)-induced murine hepatocellular carcinoma (HCC) samples.
Total RNA was isolated from 6 HCC specimens, which represented a different set of samples from those shown in Figure 1e. They were subjected to quantitative reverse-transcription (RT)-PCR analysis to compare the expression levels of *Hes1* and those of *Delta-like 1* (*Dll1*), *Delta-like 4* (*Dll4*), *Jagged-1* (*Jag1*), and *Jagged-2* (*Jag2*).

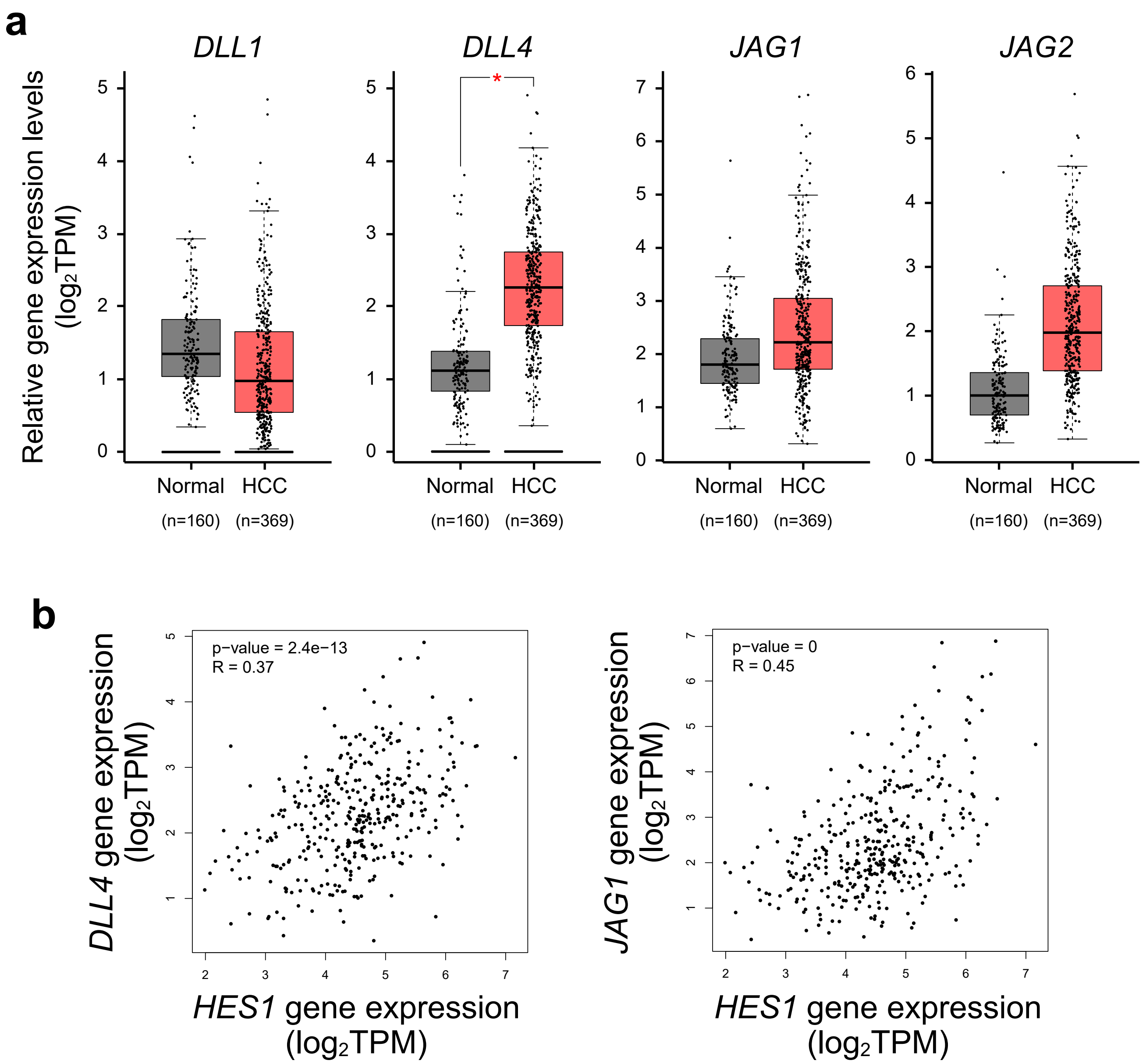

Supplementary Figure 2. Expression levels of Notch ligands in human HCC tissues.
(**a**) According to the Cancer Genome Atlas (TCGA) data, *Delta-like 4* (*DLL4*) is upregulated in human HCC tissues (n = 369) relative to normal liver tissues (n = 160). The data of other Notch ligands are also shown. An asterisk indicates that the difference between the groups is statistically significant ($P < 0.01$). (**b**) Significant correlations were observed between the amounts of *HES1* mRNAs and *DLL4* or *Jagged-1* (*JAG1*) mRNA present in the human HCC tissues. These data were obtained from the Gene Expression Profiling Interactive Analysis (GEPIA) database (http://gepia.cancer-pku.cn/index.html).

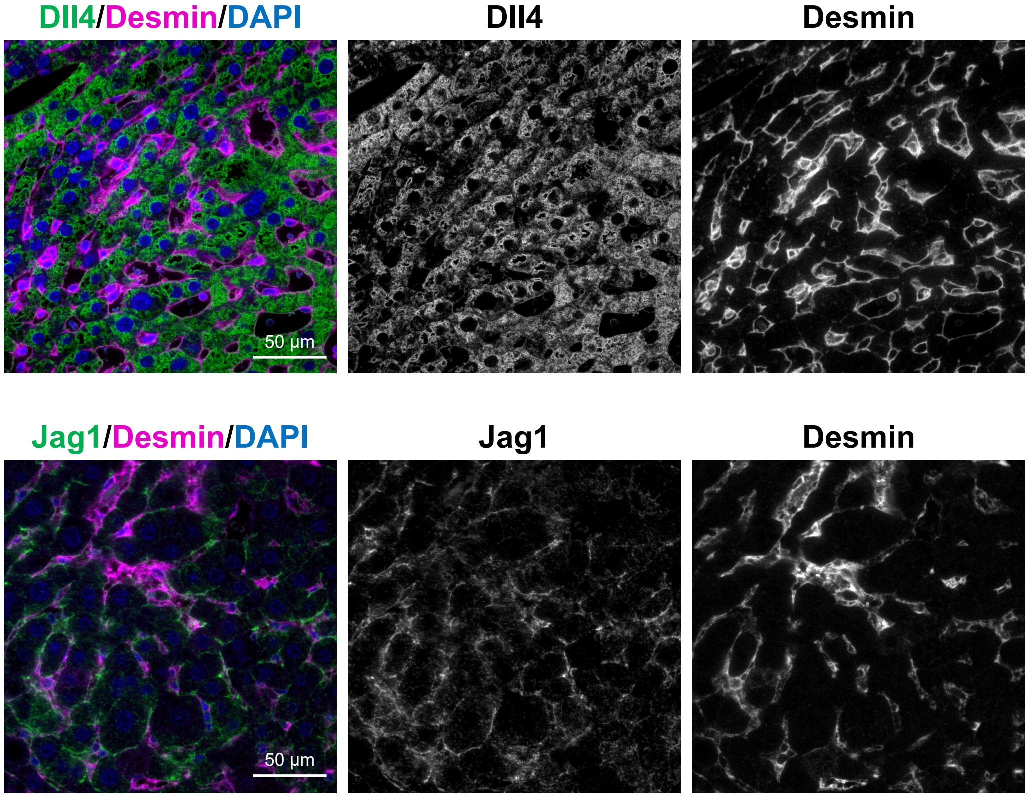

Supplementary Figure 3. Differential expression of Dll4 and Jag1 in DEN-induced murine HCC. Immunofluorescent co-staining of Dll4 or Jag1 (*green*) and desmin (*magenta*) alomg with nuclear staining with 4',6-diamidino-2-phenylindole (DAPI) (*blue*) was performed on DEN-induced HCC tissues. The *upper* and *lower* panels showed the pictures of the peripheral and intrinsic regions, respectively, within the tumor. *Scale bar*, 50 µm.

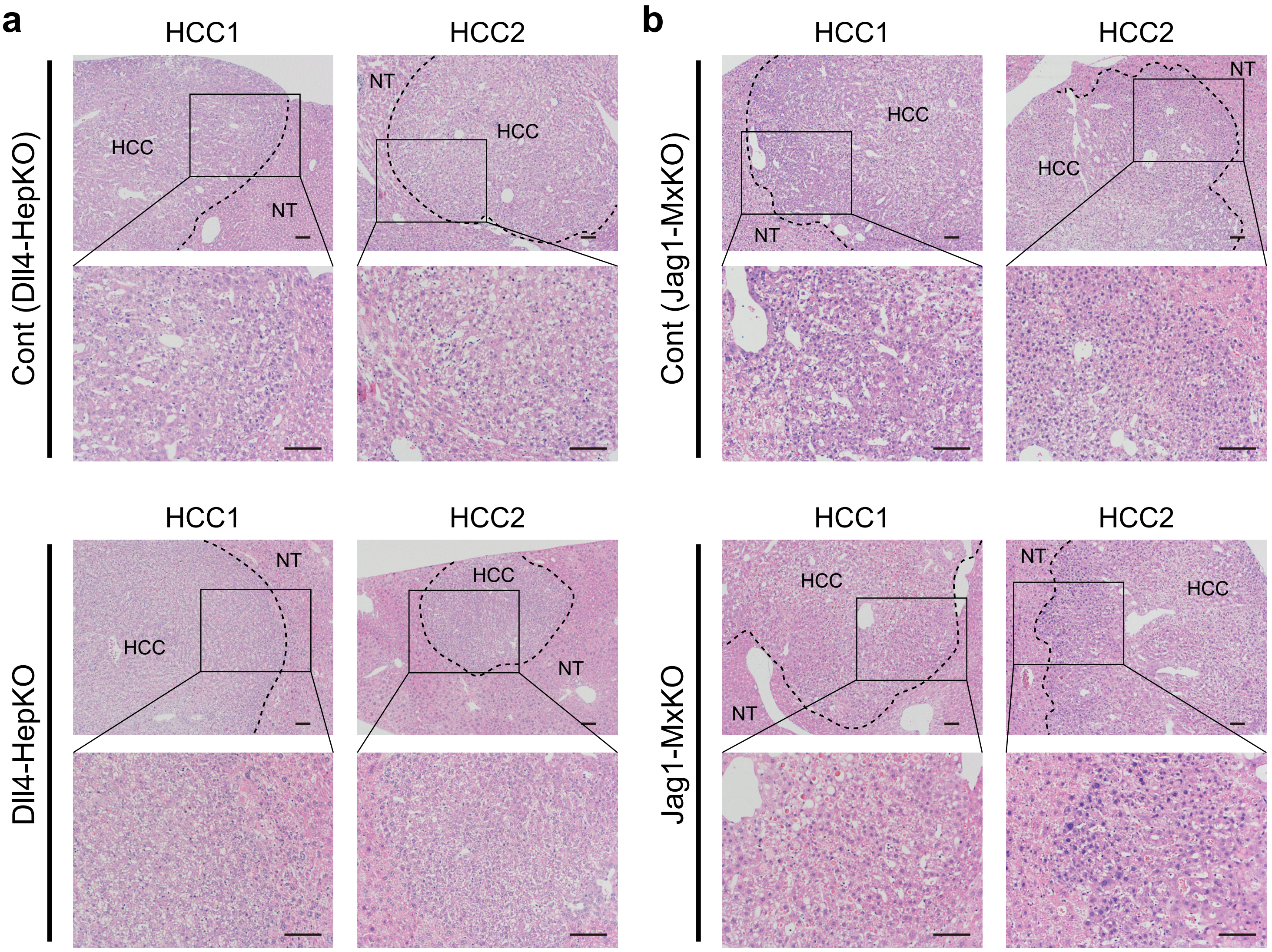

Supplementary Figure 4. Histological features of HCC tissues developed in Dll4-HepKO and Jag1-MxKO mice.

Hematoxylin and eosin staining was performed on HCC specimens from Dll4-HepKO (**a**) and Jag1-MxKO (**b**) mice together with their control animals. The border between HCC and non-tumorous tissue is indicated by a *hatched line*. *Scale bar*, 100 µm.

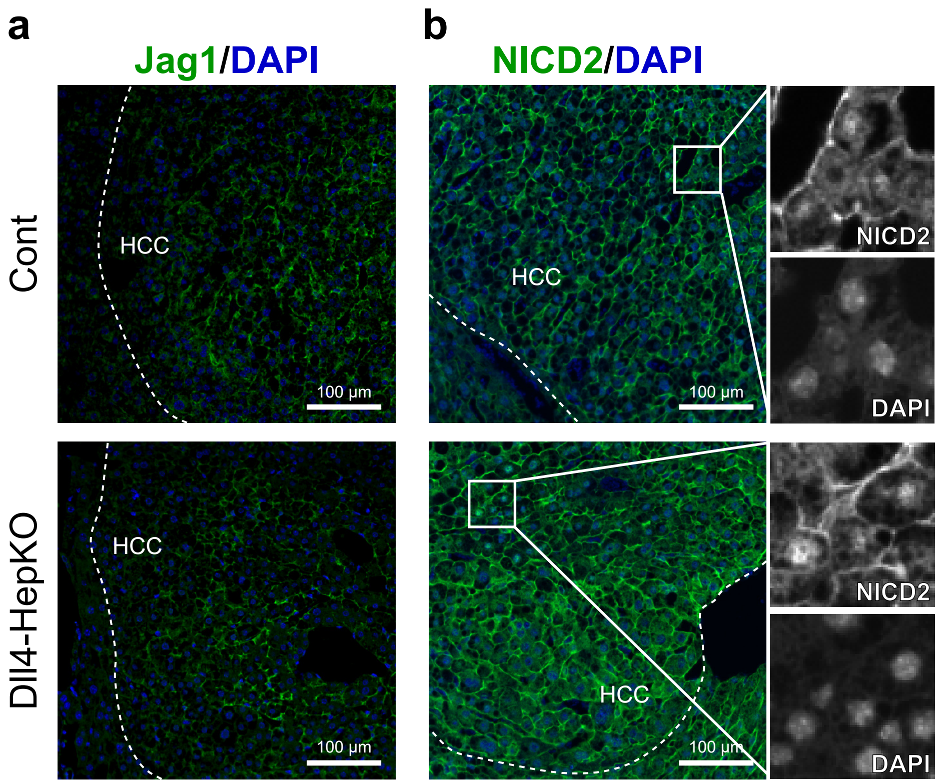

Supplementary Figure 5. Expression of Jag1 and activation status of Notch2 in cancer cells induced by *Dll4* deletion.

Immunofluorescent staining of Jag1 (**a**) or NICD2 (**b**) along with nuclear staining with DAPI was performed on DEN-induced HCC tissues in control (*Cont*) and Dll4-HepKO mice. The border between HCC and non-tumorous tissue is indicated by a *hatched line*. *Scale bar*, 100 µm.

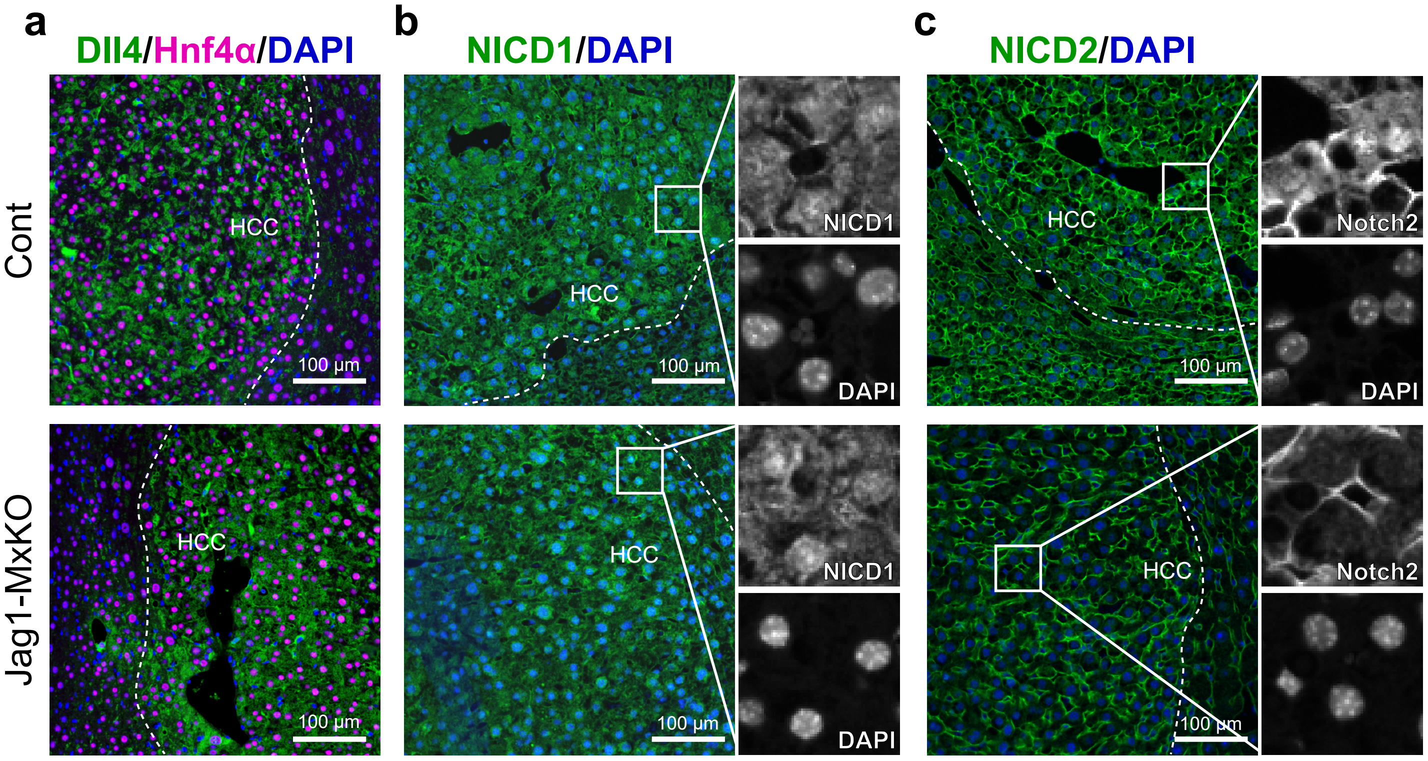

Supplementary Figure 6. Expression of Dll4 and activation status of Notch1/Notch2 in cancer cells induced by *Jag1* deletion.

(**a**) Immunofluorescent co-staining of Dll4 (*green*) and Hnf4α (*magenta*) or (**b&c**) single staining of NICD1 (**b**, *green*) or NICD2 (**c**, *green*) along with nuclear staining with DAPI (*blue*) was performed on DEN-induced HCC tissues in control (*Cont*) and Jag1-MxKO mice. The border between HCC and non-tumorous tissue is indicated by a *hatched line*. *Scale bar*, 100 µm.

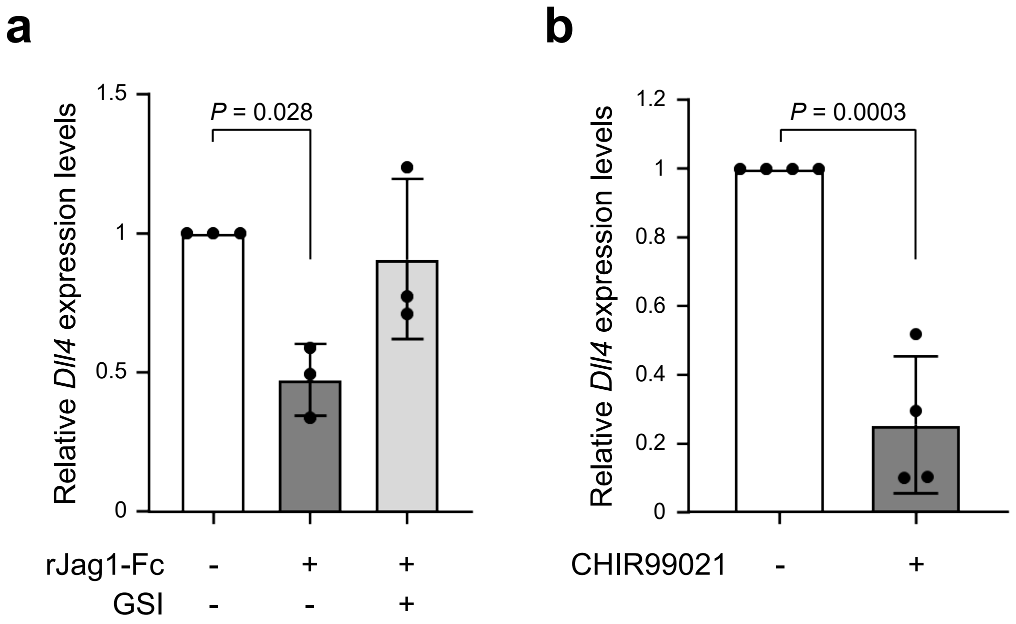

Supplementary Figure 7. Effects of Notch and Wnt signaling on the expression levels of *Dll4* in the primary cultures of hepatocytes.

(**a**) Primary hepatocytes isolated from wild-type mice were cultured on a type I collagen-coated dish with or without further coating with recombinant human Jagged-1 Fc chimera protein (rJag1-Fc). They were treated with dimethyl sulfoxide (DMSO) or 10 μM gamma secretase inhibitor IX (GSI), a Notch signal inhibitor, for 24 h. (**b**) The same hepatocytes were cultured on a collagen-coated dish and treated with DMSO or 10 μM CHIR99021, a Wnt signal agonist, for 24 h. Total RNA was isolated from these cells and subjected to quantitative real-time PCR. The values represent the mean ± standard deviation (SD) from 3 male mice (**a**) or 4 male mice (**b**) and are expressed relative to those in control cells treated with DMSO (set as 1.0).

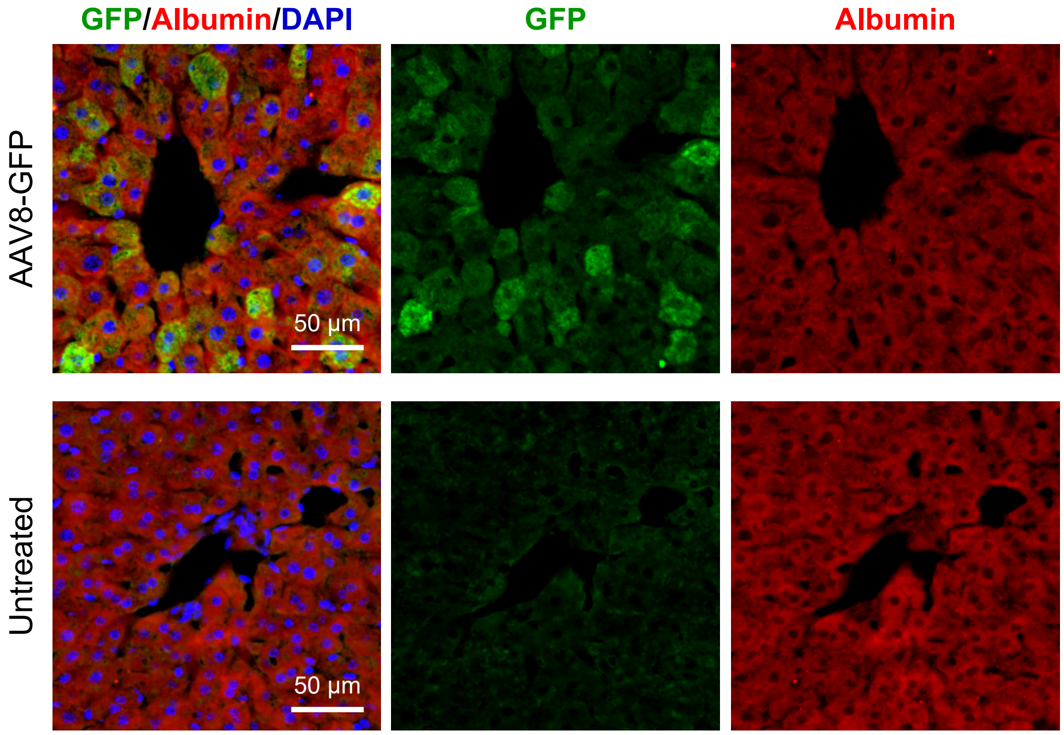

Supplementary Figure 8. Efficient infection of AAV8 to hepatocytes in the liver.
Liver specimens were obtained from mice on day 5 after intraperitoneal injection of control adeno-associated virus serotype 8 (AAV8) expressing GFP. They were subjected to immunofluorescent co-staining of GFP (*green*) and albumin (*red*, a hepatocyte marker molecule). Nuclei were stained with DAPI (*blue*). *Scale bar*, 50 µm.

Supplementary Table 1. Primary antibodies.

Target	Host	Dilution	Catalog No.	Supplier
Hes1	Rabbit	1/500*	11988	Cell Singling Technologies, Danvers, MA.
Hnf4α	Goat	1/100	sc-6556	Santa Cruz Biotechnology, Dallas, TX.
Dll4	Rabbit	1/300*	ab217860	Abcam, Cambridge, UK.
Jag1	Rabbit	1/200*	2620	Cell Singling Technologies, Danvers, MA.
Notch1 (NICD1)	Rat	1/200*	bTAN 20	Developmental Studies Hybridoma Bank (DSHB), IA.
Notch2 (NICD2)	Rat	1/200*	C651.6DbHN	Developmental Studies Hybridoma Bank (DSHB), IA.
Ki67	Rat	1/100	652402	BioLegend, San Diego, CA.
GST-p	Rabbit	1/300	311	MBL, Tokyo, Japan
Desmin	Mouse	1/100	M760	Dako, Glostrup, Denmark.
GFP	Rabbit	1/100	ab290	Abcam, Cambridge, UK.
Albumin	Goat	1/200	A90-134A	Bethyl Laboratories, Montgomery, TX.

*The TSA system (PerkinElmer, Waltham, MA) was used according to the manufacturer's instructions.

Supplementary Table 2. Secondary antibodies.

Conjugate	Target	Dilution	Catalog No.	Supplier
Alexa Fluor 488	Mouse IgG	1/300	A31619	Molecular Probes, Eugene, OR.
Alexa Fluor 555	Goat IgG	1/300	A21432	Molecular Probes, Eugene, OR.
Alexa Fluor Plus 647	Mouse IgG	1/300	A32728	Molecular Probes, Eugene, OR.
Alexa Fluor 647	Rabbit IgG	1/300	A32795	Molecular Probes, Eugene, OR.
Alexa Fluor 647	Rat IgG	1/300	A21247	Molecular Probes, Eugene, OR.
HRP (ImmPress)	Rabbit IgG	*	MP-7401	Vector, Burlingame, CA.
HRP (Simple stain)	Rat IgG	*	414311	Nichirei Bioscience, Tokyo, Japan.

*Ready to use.

Supplementary Table 3. Primers used for quantitative RT-PCR.

Target	Forward/Reverse	Sequence
Hes1	Forward	(5')- GGCCTCTGAGCACAGAAAGT-(3')
	Reverse	(5')-ATGCCGGGAGCTATCTTTCT-(3')
Dll1	Forward	(5')-TGAGAGAGGAAGGGAGAGGAA-(3')
	Reverse	(5')-AGTGCAATGGGAACAACCAG-(3')
Dll4	Forward	(5')-TATACCTGCACCTGTCTCCCA-(3')
	Reverse	(5')-TTACAGCTGCCACCATTTCG-(3')
Jag1	Forward	(5')-GGACATTATGCCTGTGACCAG-(3')
	Reverse	(5')-GGGATGCTTCCAACTTCACAC-(3')
Jag2	Forward	(5')-ACGAGGAGGATGAAGAGCTGA-(3')
	Reverse	(5')-GGGGTCTTTGGTGAACTTGTG-(3')
