# Supplementary material for: Two distinct Notch signals, Delta-like 4/Notch1 and Jagged-1/Notch2, antagonistically regulate chemical hepatocarcinogenesis in mice
Source: Commun Biol. 2022 Jan 21;5:85. doi: 10.1038/s42003-022-03013-8 (PMC8782997; doi:10.1038/s42003-022-03013-8)
Supplement: Supplementary file 2 — Description of Additional Supplementary Files [file 42003_2022_3013_MOESM2_ESM.pdf]

Description of Additional Supplementary Files

File name: Supplementary Data 1

Description: Source data used to create graphs.
