# Supplementary material for: Two distinct Notch signals, Delta-like 4/Notch1 and Jagged-1/Notch2, antagonistically regulate chemical hepatocarcinogenesis in mice
Source: Commun Biol. 2022 Jan 21;5:85. doi: 10.1038/s42003-022-03013-8 (PMC8782997; doi:10.1038/s42003-022-03013-8)
Supplement: Supplementary file 4 — Reporting Summary [file 42003_2022_3013_MOESM4_ESM.pdf]

nature portfolio

Corresponding author(s): Yutaka Inagaki

Last updated by author(s): Dec 5, 2021

Reporting Summary

Nature Portfolio wishes to improve the reproducibility of the work that we publish. This form provides structure for consistency and transparency in reporting. For further information on Nature Portfolio policies, see our Editorial Policies and the Editorial Policy Checklist.

Statistics

For all statistical analyses, confirm that the following items are present in the figure legend, table legend, main text, or Methods section.

n/a	Confirmed	
☐	☒	The exact sample size (*n*) for each experimental group/condition, given as a discrete number and unit of measurement
☐	☒	A statement on whether measurements were taken from distinct samples or whether the same sample was measured repeatedly
☐	☒	The statistical test(s) used AND whether they are one- or two-sided<br>*Only common tests should be described solely by name; describe more complex techniques in the Methods section.*
☒	☐	A description of all covariates tested
☒	☐	A description of any assumptions or corrections, such as tests of normality and adjustment for multiple comparisons
☐	☒	A full description of the statistical parameters including central tendency (e.g. means) or other basic estimates (e.g. regression coefficient) AND variation (e.g. standard deviation) or associated estimates of uncertainty (e.g. confidence intervals)
☐	☒	For null hypothesis testing, the test statistic (e.g. *F*, *t*, *r*) with confidence intervals, effect sizes, degrees of freedom and *P* value noted<br>*Give P values as exact values whenever suitable.*
☒	☐	For Bayesian analysis, information on the choice of priors and Markov chain Monte Carlo settings
☒	☐	For hierarchical and complex designs, identification of the appropriate level for tests and full reporting of outcomes
☒	☐	Estimates of effect sizes (e.g. Cohen's *d*, Pearson's *r*), indicating how they were calculated

Our web collection on statistics for biologists contains articles on many of the points above.

Software and code

Policy information about availability of computer code

Data collection	BZ-II Analyzer Ver. 2.2 (Keyence)<br>StepOne Software v2.3 (Thermo Fisher Scientific)<br>Microsoft Excel 2013 Windows (Microsoft)<br>Agilent feature extraction software v11.5.1.1 (Agilent)
Data analysis	ImageJ 1.53e (NIH)<br>GIMP 2.10.22 (GIMP)<br>GraphPad Prism 8 (GraphPad Prism Inc.)<br>GeneSpring GX software v14.9 (Agilent)

For manuscripts utilizing custom algorithms or software that are central to the research but not yet described in published literature, software must be made available to editors and reviewers. We strongly encourage code deposition in a community repository (e.g. GitHub). See the Nature Portfolio guidelines for submitting code & software for further information.

March 2021

Data

Policy information about availability of data

All manuscripts must include a data availability statement. This statement should provide the following information, where applicable:

- Accession codes, unique identifiers, or web links for publicly available datasets
- A description of any restrictions on data availability
- For clinical datasets or third party data, please ensure that the statement adheres to our policy

The data that support the findings of this study are available from the corresponding author upon reasonable request.

Field-specific reporting

Please select the one below that is the best fit for your research. If you are not sure, read the appropriate sections before making your selection.

[x] Life sciences   [ ] Behavioural & social sciences   [ ] Ecological, evolutionary & environmental sciences

For a reference copy of the document with all sections, see nature.com/documents/nr-reporting-summary-flat.pdf

Life sciences study design

All studies must disclose on these points even when the disclosure is negative.

Sample size	Sample size of sufficient statistical power were chosen based on previously published studies using similar analysis (Nakano Y, et al. Hepatology 2019; Nakano Y, et al. Hepatol Commun 2017; Nakano Y, et al. Genes Cells 2015)
Data exclusions	No individual samples, animals or data were excluded from the analyses.
Replication	For each series of experiments, attempts at replication were successful. A statement describing experimental replication is included in the Methods section and the legend to each figure.
Randomization	Mice were randomly assigned to each experiment.
Blinding	Researchers were not blinded when analyzing results.

Reporting for specific materials, systems and methods

We require information from authors about some types of materials, experimental systems and methods used in many studies. Here, indicate whether each material, system or method listed is relevant to your study. If you are not sure if a list item applies to your research, read the appropriate section before selecting a response.

Materials & experimental systems

n/a	Involved in the study	
[ ]	[x]	Antibodies
[ ]	[x]	Eukaryotic cell lines
[x]	[ ]	Palaeontology and archaeology
[ ]	[x]	Animals and other organisms
[x]	[ ]	Human research participants
[x]	[ ]	Clinical data
[x]	[ ]	Dual use research of concern

Methods

n/a	Involved in the study	
[x]	[ ]	ChIP-seq
[x]	[ ]	Flow cytometry
[x]	[ ]	MRI-based neuroimaging

Antibodies

Antibodies used

Hes1 Rabbit 1/500* 11988 Cell Singling Technologies, Danvers, MA.
Hnf4α Goat 1/100 sc-6556 Santa Cruz Biotechnology, Dallas, TX.
Dll4 Rabbit 1/300* ab217860 Abcam, Cambridge, UK.
Jag1 Rabbit 1/200* 2620 Cell Singling Technologies, Danvers, MA.
Notch1 Rat 1/200* bTAN 20 Developmental Studies Hybridoma Bank (DSHB), IA.
Notch2 Rat 1/200* C651.6DbHN Developmental Studies Hybridoma Bank (DSHB), IA.
Ki67 Rat 1/100 652402 BioLegend, San Diego, CA.
GST-p Rabbit 1/300 311 MBL, Tokyo, Japan
Desmin Mouse 1/100 M760 Dako, Glostrup, Denmark.
GFP Rabbit 1/100 ab290 Abcam, Cambridge, UK.

	Albumin Goat 1/200 A90-134A Bethyl Laboratories, Montgomery, TX.
Validation	All antibodies are commercially available and validation is provided in the data sheets of the manufacturer.

Eukaryotic cell lines

Policy information about cell lines

Cell line source(s)	AAVpro293T cells were purchased from Takara Bio (Otsu, Japan).
Authentication	The cell line was authenticated by the supplier.
Mycoplasma contamination	The cell line was not tested for mycoplasma contamination.
Commonly misidentified lines (See ICLAC register)	N/A

Animals and other organisms

Policy information about studies involving animals; ARRIVE guidelines recommended for reporting animal research

Laboratory animals	All animals used in the present study received humane care, and the experiments were approved by the Animal Experimentation Committee of Tokai University.<br>Dll4-floxed knock-in mice (Hozumi K, et al. J Exp Med 2008)<br>Jag1-floxed knock-in mice (Estrach S, et al. Development 2006)<br>Mx-Cre transgenic mice (Kuhn R, et al. Science 1995)<br>Rosa26-CreER knock-in mice (Badea TC, et al. J Neurosci 2003)
Wild animals	Wild-type C57BL/6J mice were purchased from CLEA Japan (Tokyo, Japan) and used at 8 to 10 weeks of age.
Field-collected samples	The study did not involve samples collected from the field.
Ethics oversight	Animal Experimentation Committee of Tokai University School of Medicine

Note that full information on the approval of the study protocol must also be provided in the manuscript.
